# Knowledge, experiences, and practices on safe opioid use in patients recently discharged from hospitals in Western Nepal: a qualitative study

**DOI:** 10.3389/fphar.2025.1572968

**Published:** 2025-10-01

**Authors:** Sundar Adhikari, Roshani Gurung, Shakti Shrestha, Alian A. Alrasheedy, Vibhu Paudyal, Ramesh Lamshal, Pratima Sharma, Bhuvan KC

**Affiliations:** ^1^ Department of Pharmacy, Fishtail Hospital and Research Center Pvt. Ltd., Pokhara, Nepal; ^2^ Pharmacy Program, Gandaki University, Pokhara, Nepal; ^3^ School of Pharmacy and Pharmaceutical Science, Dutton Park Campus, The University of Queensland, Woolloongabba, QLD, Australia; ^4^ Department of Pharmacy Practice, College of Pharmacy, Qassim University, Qassim, Saudi Arabia; ^5^ Florence Nightingale Faculty of Nursing, Midwifery and Palliative Care, King’s College London, London, United Kingdom; ^6^ Department of Nursing, Fishtail Hospital and Research Center Pvt. Ltd., Pokhara, Nepal; ^7^ Discipline of Pharmacy, School of Clinical Sciences, Queensland University of Technology, Brisbane, QLD, Australia

**Keywords:** opioid use, post-discharge care, pain management, patient education, low-and middle-income countries (LMICs)

## Abstract

**Background:**

Rational prescribing and use of opioids is key to managing pain and mitigating adverse effects. Limited research exists on patient’s knowledge and experiences regarding use of prescribed opioid use within low resource settings.

**Objectives:**

This study aimed to explore the knowledge, experiences, and practices related to opioid use among patients discharged from hospitals in Western Nepal.

**Methods:**

A qualitative study was undertaken involving semi-structured interviews with patients discharged from a tertiary hospital in Western Nepal who had been prescribed opioids for pain management. Thematic analysis was used to identify key themes related to opioid use, counseling practices, and knowledge gaps.

**Results:**

A total of 30 patients were interviewed. Analysis showed five themes *viz.* awareness of purpose, effectiveness and adverse effects, patient experience with opioid therapy, adherence to administration instruction and storage practices, risk awareness and safety practices, and follow-up and continuity of care. Participants showed awareness of opioid dosage, frequency, and storage but lacked knowledge of adverse effects, dependence, and tapering. Counselling from healthcare providers was noted but was often limited and inconsistent in regard to adverse effects and the long-term risks. None of the participants reported having a shared plan of action including follow up post-discharge.

**Conclusion:**

There are critical gaps in patient education and discharge counselling regarding opioid use in Nepal. Strengthening pharmacist-led counselling, implementing structured discharge protocols, and developing tailored educational interventions are essential to promote safe opioid use and reduce misuse in low resource settings.

## 1 Introduction

Opioids are widely recognised as an essential analgesic for managing short-term pain during acute painful events, such as surgical procedures, trauma, and medical interventions and palliative and cancer-related pain (International Association for the Study of Pain, 2024). However, the therapeutic benefits of opioids are often outweighed by their potential for misuse, abuse and adverse effects. In 2019, the World Health Organization (WHO) reported 600,000 deaths attributed to drug use globally, with opioids contributing to 80% of these fatalities (World Health Organizati on, 2025). The overuse, misuse and increasing rates of opioid-related abuse pose significant public health challenges, affecting health systems and societies worldwide. There is a significant gap in knowledge regarding the safe and effective use of opioids in low and middle-income countries (LMICs), including Nepal (Jadhari et al., 2024; Biancuzzi et al., 2022; Brady et al., 2016; Kharasch et al., 2022; Bryson and Boxhorn, 2023). Limited research exists on the rational use of prescription opioids, as well as their dispensing practices and utilisation by consumers in these settings.

In Nepal, opioids such as morphine, fentanyl, pethidine, codeine and tramadol are commonly used for managing acute pain, cancer-related pain and palliative care (Shrestha, 2025; Ministry of Health and Population (MoHP-Nepal), 2021). Recognizing their significance, the Nepalese List of Essential Medicine (2001) enlists several opioids as essential for pain management and palliative care (Shrestha, 2025; Ministry of Health and Population MoHP-Nepal, 2021). Recent studies from Nepal show that opioids are becoming more accessible in hospital settings, where they are increasingly recognized as part of standards of care in pain management (Cleary et al., 2013; Ghimire et al., 2023; Chaudhary et al., 2022). However, outside tertiary care settings, there is a lack of data on how prescription opioids are utilised by patients at households’ level once they are discharged from the tertiary care centres. Additionally, there is a lack of research on patients’ knowledge of opioids and their ability to use them appropriately at home. Previously, Jadhari et al., highlighted that restricted access to healthcare resources, together with low health literacy, heightens the risk of diversion, misuse and abuse of opioids in LMICs (Cleary et al., 2013; Ghimire et al., 2023; Chaudhary et al., 2022).

This study addresses these gaps by qualitatively exploring patients’ knowledge and experiences with opioid use during transitions of care from hospital to community settings in Western Nepal. By examining these dynamics, this research seeks to provide insights into safe and rational use of opioids in LMIC contexts.

## 2 Materials and methods

### 2.1 Study design and setting

This study uses qualitative methods based on semi-structured interviews. It was conducted with patients who were discharged with at least one opioid prescription from a tertiary care hospital in Western Nepal.

The study site is a 100-bed tertiary care hospital in Western Nepal with 200 inpatient admissions weekly. This tertiary care hospital provides 24-h healthcare services, including pain management.

### 2.2 Participants and recruitment

This hospital provides comprehensive inpatient care to a diverse patient population from Western Nepal, offering a wide range of treatments and surgical procedures. It was chosen for this study due to its specialized inpatient services and surgical facilities, which cater to a varied demographic from the region.

Convenience sampling was employed due to limited resources and to maximize participation within the discharge period. Patients were eligible for the study if they were over 18 years of age, had received at least one opioid prescription, and were willing to participate. Those with cognitive impairments or language barriers were excluded to ensure informed consent and data reliability.

A diverse sample of participants, encompassing varying demographics and discharged medical conditions, was included in the study to capture a range of viewpoints.

Each participant received a gift box containing essential items (masks and sanitizer) as a token of appreciation for their time and participation. The gift was provided irrespective of participants’ responses, and all participants were informed that participation was voluntary and that honest experiences and opinions were most valuable. Participants could withdraw at any time without any consequences.

### 2.3 Development of interview guide

An interview guide was developed by members of the research team through a comprehensive literature review on hospital opioid use, opioid use upon discharge, and patients’ perceptions of opioids, with a particular focus on LMICs like Nepal. The guide was refined through discussions with clinical pharmacists, nurses, and public health researchers. It was further revised for clarity through a pilot test with three patients recently discharged from hospitals who had received opioids.

The finalized interview questionnaire was translated from English to Nepali by two research team members (BKC and SA). The questionnaire was then reviewed by a bilingual professional translator with a public health background.

The interview guide (Supplementary Table 1) included questions and follow-up prompts exploring discharged patients’ knowledge, experiences, and practices regarding the safe, effective, appropriate, and judicious use of opioids, including issues related to adverse effects, tapering, and dependence.

### 2.4 Data collection and analysis

Face-to-face semi-structured interviews were carried out to collect the data, with an average duration of approximately 25 min.

A total of 70 patients were approached for the study. Of these, 20 declined without providing a reason, 15 declined due to being busy, and 5 were excluded due to cognitive impairment, resulting in a final sample of 30 participants. While this sample does not represent all patients discharged with opioid prescriptions from our study site hospital, it included diverse demographics, medical conditions, and opioid experiences to capture a range of perspectives. We acknowledge that convenience sampling may introduce selection bias, as participants who agreed to take part might differ from those who declined in terms of health literacy, attitudes toward opioids, or engagement with healthcare providers.

Interviews were conducted until data saturation was reached, meaning no new themes or codes emerged from additional participants. Saturation was assessed iteratively during the coding process: after each set of 5–6 interviews, transcripts were independently coded by two researchers (BKC and RG), and codes were compared and discussed within the research team (BKC, SA, RG, AAA). When three consecutive interviews did not generate new codes or themes, saturation was considered achieved. Recruitment was concluded at 30 participants, as data saturation had been reached. We also recognize that approaching a larger number of participants could have provided additional perspectives, but resource limitations constrained recruitment.

All interviews were audio-recorded and transcribed verbatim in Nepali by SA and RG. Transcripts were read back to participants for validation, allowing them to correct or remove any content that did not accurately reflect their input. The finalized transcripts were then translated into English by BKC and SA and reviewed by a professional translator.

With participants’ consent, prescriptions from all 30 participants were collected and analyzed to gather information on both opioid and non-opioid analgesics. A simple descriptive analysis of the collected prescription data was then performed.

### 2.5 Thematic analysis

Both deductive and inductive coding approaches were used in the thematic analysis. Deductive coding was guided by the research questions and predefined areas of investigation, while inductive coding explored emerging themes raised by patients, enabling the identification of new themes beyond the predefined framework (Olsen et al., 2023).

The final coding framework included seven overarching themes, each with sub-themes and specific codes (Supplementary Tables 1, 2. Final coding framework), covering areas such as understanding of opioid prescription, safe use and storage, communication and counselling, awareness of adverse effects, concerns about dependence and tolerance, lifestyle and safety precautions, and patient experience and satisfaction.

All interview transcripts were independently coded by two members of the research team (BKC and SA). After each set of 5–6 interviews, codes were compared and discussed within the research team (BKC, SA, RG, AAA) to assess whether new codes or themes emerged. Saturation was considered achieved when three consecutive interviews did not generate new codes or themes across the framework. Intercoder agreement was established through regular consensus discussions rather than through a formal statistical measure such as Kappa, which is common in qualitative research. To enhance rigor and minimize potential bias, all codes and themes were additionally peer-reviewed by an independent qualitative researcher not involved in the original analysis, and illustrative quotes were mapped to each code to ensure transparency and confirmability. These were merged in the final analysis into five main themes. The final themes were arranged to reflect both overarching patterns in the data and the perspectives shared by participants, ensuring a comprehensive exploration of the issues surrounding opioid use, patient experiences, and associated outcomes.

### 2.6 Ethics

Ethical approval for this research project was obtained from the National Health Research Council (NHRC) Ethical Review Board (Ref. No. 1080) and the hospital authority. Each participant provided a written consent prior to their involvement in this study, which also included consent to audio-record the interview.

## 3 Results

### 3.1 Demographics

There were 30 participants in this study. Half of the participants as seen in Table 1 were males (n = 15), and the majority were aged 20–39 years (n = 18). The mean age of participants was 37.9 years. The average interview length was 25 min.

**TABLE 1:** Participants demographics.

Demographic attribute	Category	Frequency	Percentage (%)
Sex	Male	15	50.0
	Female	15	50.0
Marital Status	Married	20	66.7
	Unmarried	10	33.3
Age Group (Years)	20–29	6	20.0
	30–39	12	40.0
	40–49	8	26.7
	≥50	4	13.3

### 3.2 Opioid use

As seen in Table 2, most patients were prescribed the combination of codeine and paracetamol, followed by tramadol 50 mg and codeine 15 mg. The majority of prescriptions included “when needed” instructions. The longest duration of opioid use was for morphine 10 mg, prescribed at night for 1 month, while the shortest duration was 3 days for several other medications see Table 3.

**TABLE 2:** Opioid type and dosage frequency (N = 36 medicines, based on 30 prescription).

Medication	Dosage frequency	Total prescriptions (N = 36)	Percentage
Codeine phosphate + Paracetamol 500 mg	2–3 times/day	14	38.9%
Tramadol 50 mg	2–3 times/day	11	30.6%
Codeine 15 mg	2–3 times/day	6	16.7%
Morphine 10 mg	At bedtime/2 times/day	3	8.3%
Tramadol + Paracetamol	As needed	1	2.8%
Morphine syrup 10mg/5 mL	At bedtime	1	2.8%

N = number of medicines.

**TABLE 3:** Duration of opioid prescription (N = 36 medicines, based on 30 prescription).

Duration prescribed	Frequency	Percentage
3 days	22	61.1%
5 days	9	25.0%
7 days	2	5.6%
>7 days/PRN	3	8.3%

### 3.3 Themes

Five main themes were identified: education and awareness on opioid use, adherence to administration instruction and storage practices, risk awareness and safety practices, follow-up and continued care, and patient experience with opioid therapy.

#### 3.3.1 Awareness of purpose, effectiveness and adverse effects

Participants showed varying levels of knowledge regarding opioid use. Those with prior experience using opioids were generally more informed about their intended purpose, common adverse effects, and proper management. However, many participants struggled to differentiate opioids from other pain medications or understand their broader implications.

*“Yes, I know why opioids analgesic … (was) … prescribed … (for)… me for pain relief … because I … (have)… a … (complex) … pain … they … also tell me about … constipation … making sleepy … that’s all I can remember”* #Pt30

*No, I don’t know about this medicine (…). …I was only told that among these 3 medicines … one is for pain…. (shows all three medicines) … I do not know what this medicine actually does … … or what I need to do … or not do … while using …. (this)… medicine…*#Pt17

Pharmacists and doctors were identified as the primary sources of information. Both written and verbal instructions were helpful for enhancing knowledge of indications and appropriate use. However, participants’ knowledge beyond pain relief properties was often limited.

“Yes, I know. Some are 3 times, some one time according to the prescription. It is written*…* by pharmacist…” #Pt30

“I do not remember much….they told me it is a strong medicine and can cause some effect, ….but I can’t remember anything other than headache…

(probe*…*)

No one told me about using tools or riding bike etc … but I know that alcohol cannot be taken while taking medicines*…* Dr. asked me at the time of admission if I smoke, drink alcohol or not….” Pt#3

#### 3.3.2 Patient experience with opioid therapy

Participants’ experiences ranged from satisfaction with pain relief to confusion and misconceptions about opioids. Some expressed gratitude for pain control, while others were unclear about the clinical purpose or long-term effects. Misconceptions included associating opioids exclusively with severe pain or uncertainty about home use.

“I didn’t know its which class…. I now ….know*…* it’s … for pain. During hospital stay I told I have pain….and they gave (injected) this medicine for me … …. after that pain relieved….so far good.” (#Pt8)

“I was not having pain. My hospital stay was nice. Thank you all for your care … Now they have given this for home … I do not know … how it works … I only know I have to … take it on time … that’s all.” (#Pt13)

“I don’t know about it. I only know that I … should not … use it … as per my wish … I … Only should use if I have very high amount of pain.” (#Pt28)

#### 3.3.3 Adherence to administration instruction and storage practices

Most participants were aware of the correct dosage, frequency, and administration instructions, including timing with meals. However, some were confused about the duration or PRN dosing, and none reported receiving tapering plans after pain subsided.

“Yes, I know how to take this … 2 times daily for 3 days. And then only if necessary.” (#Pt11)

“I will have to see the marking on the label to know when to take the medicine … for how long … I do not remember … I will take those till I come to see the doctor next week.” (#Pt16)

“I think I can stop it when the pain comes down … I will just stop it like that … they told me to use it till the pain is there.” (#Pt29)

Safe storage practices were generally acknowledged, such as keeping opioids out of children’s reach, though detailed knowledge of secure storage was inconsistent.

“Keeping it out of reach of children. To be kept only in the area which I have access. I should use it at bedtime so I will keep it in bed.” (#Pt3)

#### 3.3.4 Risk awareness and safety practices

Participants recalled common side effects, including nausea, dizziness, and constipation, but none mentioned serious effects such as sedation or respiratory depression, indicating gaps in communication or retention. Awareness of dependence and tolerance varied: some recognized potential risks, while others had little knowledge.

“Yes, I was told not to drive and take alcohol … because that medicine may make me feel a bit drowsy.” (#Pt1)

“Slightly, because the pharmacist told me to stop the new medicine after 3 days. So, I thought this medicine may develop … dependency.” (#Pt27)

“No, I do not have much idea regarding the other effects of this medicine … like making it a habit … if I take it for a long time.” (#Pt11)

#### 3.3.5 Follow-up and continuity of care

Participants acknowledged the importance of follow-up for pain management, with many planning to monitor pain and consult doctors if pain persisted. Pharmacists’ guidance was emphasized as critical for safe opioid use, including adherence, discontinuation, and monitoring for dependency. However, clarity on when to seek consultation or the scope of follow-up care was limited, indicating potential gaps in patient education.

“Yes, Dr. told me for the visit in 7 days. We will decide about the pain management and future procedures too.” (#Pt12)

“I will take the medicine as Dr. told and will check again if the pain is still there … but I also think it’s better not to take too much because pharmacist said to stop it early if I feel better.” (#Pt18)

## 4 Discussion

### 4.1 Summary of findings

This qualitative study highlights significant gaps in appropriate opioid use among patients discharged from hospitals in Western Nepal, particularly in dispensing and monitoring practices. While patients demonstrated some awareness of opioid dosage and storage, their knowledge of adverse effects, tolerance, dependence, tapering and post-discharge monitoring was limited. These findings underscore critical challenges in opioid management and the need for targeted interventions to ensure safe and effective pain management.

### 4.2 Sampling considerations and transferability

As a qualitative study, the primary aim of this study was to explore in-depth patient experiences and knowledge regarding opioid use rather than to produce statistically generalizable results. The use of a convenience sample and the skewed composition of our participants (predominantly younger patients and trauma cases) likely influenced the patterns of knowledge and experiences captured in this study. Younger patients may have different health literacy, engagement with healthcare providers, and pain management expectations compared with older or chronically ill patients. Similarly, trauma cases might have distinct opioid prescribing patterns, discharge instructions, and recovery trajectories and follow-up plan compared with patients with chronic conditions. Consequently, the perspectives identified here may not fully reflect the broader population of patients discharged with opioids in Nepal, and caution is needed when transferring these findings to other patient groups or healthcare settings.

### 4.3 Comparison with international evidence

In high-income countries (HICs), structured discharge protocols, prescription monitoring programs, and tapering guidelines are routinely implemented to reduce dependence, enhance patient safety, and promote appropriate opioid discontinuation (Dowell et al., 2016; Furlan et al., 2010; Berna et al., 2015; Briggs, 2012; Therapeutic Goods Administration TGA, 2021; Tenni et al., 2023; Davis et al., 2020; World Health Organizati on, 2025). The CDC opioid prescribing guideline (Dowell et al., 2016; Furlan et al., 2010; Berna et al., 2015; Briggs, 2012; Therapeutic Goods Administration TGA, 2021; Tenni et al., 2023; Davis et al., 2020; World Health Organizati on, 2025), the RACGP framework in Australia (Dowell et al., 2016; Furlan et al., 2010; Berna et al., 2015; Briggs, 2012; Therapeutic Goods Administration TGA, 2021; Tenni et al., 2023; Davis et al., 2020; World Health Organizati on, 2025), and WHO (Dowell et al., 2016; Furlan et al., 2010; Berna et al., 2015; Briggs, 2012; Therapeutic Goods Administration TGA, 2021; Tenni et al., 2023; Davis et al., 2020; World Health Organization, 2025) recommendations emphasise counselling, monitoring, and tapering as core components of safe opioid use. Similarly, evidence from other LMICs shows that structured education and follow-up can mitigate misuse and dependence risks even in resource-limited settings (Jadhari et al., 2024; Seangrung et al., 2021; Hassan et al., 2024). These practices stand in contrast to the current Nepalese context, where discharge protocols are less formalised and post-discharge monitoring is absent.

### 4.4 Explanation of divergences

The divergences observed in our study can be explained by systemic and contextual factors. Nepal lacks prescription monitoring systems, pharmacist-led discharge counselling, and standardised opioid tapering guidelines and frameworks that are well established in HICs (Berna et al., 2015; Briggs, 2012; Therapeutic Goods Administration TGA, 2021; Tenni et al., 2023; Davis et al., 2020; Shrestha et al., 2022; Shrestha et al., 2025). In LMICs like Nepal, resource constraints and workforce shortages further limit opportunities for structured patient education and follow-up, particularly after surgical or trauma-related admissions (Shah et al., 2021; Kruk et al., 2018). In addition, the sample in this study was skewed towards younger trauma patients, who are more likely to receive short-term prescriptions and less likely to receive comprehensive counselling on tapering or long-term dependence risks. These factors collectively explain why patients had partial knowledge of opioid use but lacked awareness of tapering, dependence, and long-term risks.

### 4.5 Implications for policy and practice in Nepal

Our findings underscore the urgent need to strengthen discharge practices and post-discharge support for responsible opioid use in Nepal. Based on our study, key practical and policy implications include:

1. Developing standardized discharge counselling protocols covering appropriate use instructions, potential adverse effects, risk of dependence, and tapering guidance (Furlan et al., 2010; Berna et al., 2015; Briggs, 2012; Therapeutic Goods Administration TGA, 2021; Tenni et al., 2023; Davis et al., 2020).
2. Training doctors and pharmacists in safe opioid prescribing, tapering strategies, and patient education (Furlan et al., 2010; Seangrung et al., 2021; Hassan et al., 2024).
3. Providing patient-friendly educational materials to enhance understanding of opioid use and safety (Therapeutic Goods Administration TGA, 2021; Seangrung et al., 2021).
4. Integrating pharmacists into post-discharge follow-up care to strengthen continuity and monitoring (Seangrung et al., 2021; Hassan et al., 2024; Shrestha et al., 2022).
5. Establishing a prescription monitoring system adapted from high-income country models but tailored to be feasible within Nepal’s healthcare system (Berna et al., 2015; Shrestha et al., 2022; Shrestha et al., 2025; Shah et al., 2021; Kruk et al., 2018).

### 4.6 Strength and limitation

This study provides novel qualitative evidence on patient knowledge and experiences with opioids at discharge in Nepal, a context where little evidence exists. Several limitations should be acknowledged. First, our study is qualitative in nature and captures participants’ knowledge regarding appropriate opioid use at a point in time, so it does not observe changes over time. Second, due to the convenience nature of sampling, the sample was skewed toward younger participants and trauma cases, which may limit transferability of findings to older patients or those with chronic conditions. Third, participants who agreed to take part may differ from those who declined. Fourth, the study was conducted in a single tertiary hospital, which may limit generalizability to other healthcare settings, including rural hospitals or primary care facilities. Fifth, while a larger sample might have captured additional perspectives, recruitment was concluded at 30 participants due to resource constraints and the achievement of data saturation. Sixth, providing a gift box may have introduced potential response bias.

### 4.7 Future research

Future studies should use purposive or stratified sampling across diverse hospitals, include patients with chronic conditions, and employ longitudinal designs to track knowledge and practices over time. Further work is also needed to pilot and evaluate pharmacist-led discharge counselling and prescription monitoring systems in the Nepalese context.

## 5 Conclusion

This study identifies critical gaps in opioid education and post-discharge monitoring among patients in Western Nepal, with limited awareness of adverse effects, dependence, and tapering protocols. In contrast to high-income countries, where structured discharge and monitoring practices are standard, Nepal lacks such systems, reflecting broader systemic and resource constraints. Addressing these gaps requires the development of standardized discharge protocols, enhanced training for prescribers and pharmacists, and the provision of accessible patient education materials and follow-up of patients on opioids. Integrating pharmacists into post-discharge follow-up could further strengthen continuity of care and reduce risks of misuse and dependence. Future research should focus on adapting and testing interventions that promote appropriate opioid use among post-discharge patients, drawing on international best practices while being responsive to Nepal’s specific healthcare challenges.

## Data Availability

The raw data supporting the conclusions of this article will be made available by the authors, without undue reservation.

## References

[B1] Berna C. Kulich R. J. Rathmell J. P. (2015). Tapering long-term opioid therapy in chronic noncancer pain: evidence and recommendations for everyday practice. Mayo Clin. Proc. 90 (6), 828–842. 10.1016/j.mayocp.2015.04.003 26046416

[B3] Biancuzzi H. Dal Mas F. Brescia V. Campostrini S. Cascella M. Cuomo A. (2022). Opioid misuse: a review of the main issues, challenges, and strategies. Int. J. Environ. Res. Public Health 19 (18), 11754. 10.3390/ijerph191811754 36142028 PMC9517221

[B4] Brady K. T. McCauley J. L. Back S. E. (2016). Prescription opioid misuse, abuse, and treatment in the United States: an update. Am. J. Psychiatry 173 (1), 18–26. 10.1176/appi.ajp.2015.15020262 26337039 PMC4782928

[B5] Briggs E. (2012). Evaluating the impact of pain education: how do we know we have made a difference? Br. J. Pain 6 (2), 85–91. 10.1177/2049463712449961 26516475 PMC4590105

[B6] Bryson E. O. Boxhorn C. E. (2023). The opioid epidemic: origins, current state, and potential solutions. Cambridge: Cambridge University Press.

[B7] Chaudhary A. Sarraf D. P. Gupta A. K. KC Bhuvan (2022). Prescribing pattern of analgesics in hospitalized patients in surgical Unit at Nepalgunj medical College and Teaching hospital. J. Karnali Acad. Health Sci. 5 (2). Available online at: http://jkahs.org.np/jkahs/index.php/jkahs/article/view/682.

[B8] Cleary J. Radbruch L. Torode J. Cherny N. I. (2013). Formulary availability and regulatory barriers to accessibility of opioids for cancer pain in Asia: a report from the Global Opioid Policy Initiative (GOPI). Ann. Oncol 2013 24, xi24–xi32. 10.1093/annonc/mdt500 24285226

[B9] Davis M. P. Digwood G. Mehta Z. McPherson M. L. (2020). Tapering opioids: a comprehensive qualitative review. Ann. Palliat. Med. 9 (2), 586–610. 10.21037/apm.2019.12.10 32008341

[B10] Dowell D. Haegerich T. M. Chou R. (2016). CDC guideline for prescribing opioids for chronic pain -United States, 2016. JAMA 315 (15), 1624–1645. 10.1001/jama.2016.1464 26977696 PMC6390846

[B12] Furlan A. D. Reardon R. Weppler C. National Opioid Use Guideline Group (2010). Opioids for chronic noncancer pain: a new Canadian practice guideline. CMAJ 182 (9), 923–930. 10.1503/cmaj.100187 20439443 PMC2882451

[B13] Ghimire R. Anjil K. C. Shreewastav R. K. (2023). Evaluation of prescription pattern of analgesics at Emergency Department of tertiary care hospital: a descriptive Cross-Sectional study. J. Nobel Med. Coll. 12 (1), 17–21. 10.3126/jonmc.v12i1.56265

[B14] Hassan A. A. Ibrahim A. M. Nadkarni A. (2024). A systematic review of task-sharing interventions for substance use and substance use disorder in low- and middle-income countries. Drug Alcohol Depend. 256, 111093. 10.1016/j.drugalcdep.2024.111093 38309090

[B15] International Association for the Study of Pain (2024). IASP statements on opioids for pain management. Washington, D.C.: International Association for the Study of Pain. Available online at: https://www.iasp-pain.org/advocacy/iasp-statements/opioids-for-pain-management/.

[B16] Jadhari R. Pathak N. Shrestha R. Shrestha S. Kc B. Gan S. H. (2024). Advancing opioid stewardship in low-middle-income countries: challenges and opportunities. J. Pharm. Policy Pract. 17 (1), 2345219. 10.1080/20523211.2024.2345219 38845626 PMC11155429

[B17] Kharasch E. D. Clark J. D. Adams J. M. (2022). Opioids and public health: the prescription opioid ecosystem and need for improved management. Anesthesiology 136 (1), 10–30. 10.1097/ALN.0000000000004065 34874401 PMC10715730

[B18] Kruk M. E. Gage A. D. Arsenault C. Jordan K. Leslie H. H. Roder-DeWan S. (2018). High-quality health systems in the Sustainable Development Goals era: time for a revolution. Lancet Glob. Health 6 (11), e1196–e1252. 10.1016/S2214-109X(18)30386-3 30196093 PMC7734391

[B19] Ministry of Health and Population MoHP-Nepal (2021). National list of essential medicines Nepal. Kathmandu, Nepal: Sixth revision.

[B20] Olsen W. M. Freeman C. Adewumi A. La Caze A. (2023). A scoping review of health system guidelines for pharmacist responsibilities when dispensing opioids. Explor. Res. Clin. Soc. Pharm. 12, 100382. 10.1016/j.rcsop.2023.100382 38155917 PMC10753378

[B22] Seangrung R. Tempeetikul T. Pannarunothai S. Sakdanuwatwong S. (2021). Perspectives of pain specialists, patients, and family members on long-term opioid use for chronic non-cancer pain: a qualitative study. BMC Anesthesiol. 21, 275–0. 10.1186/s12871-021-01501-8 34753421 PMC8576950

[B23] Shah R. K. Markus A. F. Shah N. K. (2021). Tackling the challenges of providing surgical services in low resource LMICs: Shortcomings in surgical healthcare in Nepal. Surg. Pract. Sci. 6, 100114. 10.1016/j.jobcr.2021.09.019 34760615 PMC8566997

[B24] Shrestha R. (2025). Opioid availability, production and supply in Nepal. Available online at: https://www.researchgate.net/publication/371510182_Opioid_availability_production_and_supply_in_Nepal.

[B25] Shrestha R. Palaian S. Sapkota B. Shrestha S. Khatiwada A. P. Shankar P. R. (2022). A nationwide exploratory survey assessing perception, practice, and barriers toward pharmaceutical care provision among hospital pharmacists in Nepal. Sci. Rep. 12 (1), 16590. 10.1038/s41598-022-16653-x 36198682 PMC9532804

[B26] Shrestha R. Shrestha S. Shrestha S. Shrestha A. Paudel B. D. Munday D. (2025). Opioid accessibility for palliative care in Nepal: a review of achievement and remaining challenges. Indian J. Palliat. Care 31 (1), 74–78. 10.25259/IJPC_118_2024 40027980 PMC11866692

[B27] Tenni P. Gurgius M. Thompson A. (2023). A guide to deprescribing opioids. Prim. Health Tasman. Available online at: https://www.primaryhealthtas.com.au/wp-content/uploads/2023/03/A-guide-to-deprescribing-opioids.pdf.

[B28] Therapeutic Goods Administration (TGA) (2021). Clinician information Sheet on opioid analgesic tapering. Canberra: Australian Government. Available online at: https://www.tga.gov.au/sites/default/files/clinician_information_sheet_on_opioid_analgesic_tapering.pdf.

[B29] World Health Organization (2025). Opioid overdose. Geneva: WHO. Available online at: https://www.who.int/news-room/fact-sheets/detail/opioid-overdose.

