# Supplementary material for: Knowledge, experiences, and practices on safe opioid use in patients recently discharged from hospitals in Western Nepal: a qualitative study
Source: Front Pharmacol. 2025 Oct 1;16:1572968. doi: 10.3389/fphar.2025.1572968 (PMC12520870; doi:10.3389/fphar.2025.1572968)
Supplement: Supplementary file 1 [file Table1.docx]

**Structured interview guide**

| **Q#** | **Original Question** | **Revised Focus / Follow-up Prompts** | **Topic Covered** |
| --- | --- | --- | --- |
| 1 | Could you please share your understanding of why the opioid medication was prescribed for you? | *Prompt:* Can you explain how you expected it to help your pain? | Purpose of opioid use |
| 2 | Could you please explain how and when you should take the medication? | *Prompt:* What do you do if you miss a dose? | Correct administration & adherence |
| 3 | Could you please let me know the correct dosage of the opioid medication given to you? | *Prompt:* Did anyone explain how dosage could change over time? | Dosage, tapering (if applicable) |
| 4 | Could you please tell me what you know about storage of opioids to prevent unauthorized access? | *Prompt:* Have you thought about keeping it away from children or others? | Safe storage |
| 5 | Were you asked about any previous ADR (adverse drug reactions) related to the opioids and non-opioid medications prescribed? | *Prompt:* Can you describe any side effects you experienced or were concerned about? | Adverse effects |
| 6 | What information were you given about the potential side effects of the opioid? | *Prompt:* Were you told about common side effects like drowsiness or constipation? | Adverse effects |
| 7 | How familiar are you with the process of reaching out to your healthcare provider if you experience adverse effects? | *Prompt:* Who would you contact first if you had a problem? | Managing adverse effects |
| 8 | Can you share your knowledge about the signs indicating the development of tolerance or dependence on opioids? | *Prompt:* How would you know if your body was getting used to the medication? | Dependence / tolerance |
| 9 | During your interaction with the pharmacist, what specific inquiries were made about the medications, including over-the-counter drugs and supplements, that you are currently taking? | *Prompt:* Did the pharmacist discuss interactions or safe use? | Safe and judicious use |
| 10 | Were you informed about potential effects of opioids on your ability to drive or operate heavy machinery? | *Prompt:* Did this affect your daily activities? | Safety awareness |
| 11 | Initial Dosage and Reduction Plan: Starting Dosage and Tapering Instructions | *Prompt:* Did anyone explain how to gradually reduce or stop the medication? | Tapering |
| 12 | Awareness of Medication Effects | *Prompt:* What effects did you notice while taking the medication? | Adverse effects / efficacy |
| 13 | Can you elaborate on how you plan to monitor and evaluate your pain while using the medication? | *Prompt:* Did anyone advise on tracking pain or side effects? | Pain management & monitoring |
| 14 | Regarding your pain management, have you engaged in discussions with your healthcare provider, including any plans for follow-up sessions? | *Prompt:* Are there any scheduled follow-ups or checks? | Follow-up & continuity of care |
| 15 | Do you have any additional comments or experiences related to your opioid prescription or pain management during your hospital stay? | *Prompt:* Is there anything you wish you had known about opioid use, risks, or safe management? | Overall experiences, gaps in knowledge |
