# Supplementary material for: Knowledge, experiences, and practices on safe opioid use in patients recently discharged from hospitals in Western Nepal: a qualitative study
Source: Front Pharmacol. 2025 Oct 1;16:1572968. doi: 10.3389/fphar.2025.1572968 (PMC12520870; doi:10.3389/fphar.2025.1572968)
Supplement: Supplementary file 2 [file Table2.docx]

**Final coding framework**

| **Theme** | **Sub-theme** | **Codes** | **Illustrative quotes** |
| --- | --- | --- | --- |
| Understanding of Opioid Prescription | Purpose of medication | Pain relief, post-surgical pain, chronic pain management | “Opioids were prescribed for managing pain after surgery” (P#14) |
|  | Dosage and adherence | \| Frequency of intake, maximum dosage, conditional use \| \| --- \| | \| “Doctor told me 2 times a day for 5 days” (P#7) \| \| --- \| |
| Safe Use and Storage | Storage practices | Keep out of reach of children, original packaging, lack of knowledge | “We keep it in a locked cupboard” (P#10) |
|  | Prevention of misuse | For personal use only, returning unused medication | “I returned the extra tablets to the pharmacy” (P#23) |
| Communication and Counseling | Information provision | Advice from doctors, pharmacists, nurses | “The pharmacist explained about nausea and dizziness” (P#25) |
|  | Follow-up and monitoring | Scheduled follow-up visits, monitoring pain | “Doctor asked me to come back in 7 days to check wound and pain” (P#18) |
| Awareness of Adverse Effects | Common side effects | Drowsiness, constipation, nausea/vomiting | “It may cause sleepiness and constipation” (P#32) |
|  | Adverse reaction management | Seek hospital if severe effects, hydration advice | “If side effects worsen, I will go to the hospital” (P#27) |
| Concerns about Dependence and Tolerance | Awareness of risks | Fear of addiction, tolerance development, habitual use | “If I use it regularly, it may make me take it for even minor pain” (P#16) |
|  | Limited knowledge | Lack of awareness, misconceptions | “We don’t directly buy this kind of medicine, so don’t know about tolerance” (P#22) |
| Lifestyle and Safety Precautions | Alcohol interaction | Explicit prohibition, inconsistent messaging | “Doctor said not to drink alcohol with this medicine” (P#9) |
|  | Driving and work safety | Restrictions on driving, heavy machinery | “I was told not to drive because of dizziness” (P#12) |
| Patient Experience and Satisfaction | Positive experiences | Effective pain relief, trust in healthcare team | “Opioids helped me sleep well after surgery” (P#5) |
|  | Negative experiences | Side effects, request for stronger medication | “Sometimes pain was still there, so I asked for stronger medicine” (P#21) |
